# P-2053. Enhancing Diabetic Foot Ulcer Outcomes: A Multifaceted Initiative at a Safety-Net Wound Care Clinic

**DOI:** 10.1093/ofid/ofaf695.2217

**Published:** 2026-01-11

**Authors:** Yuriko Fukuta, Judy C Amaya, Dora Romo Glaser, Nikki Lynn Lee, Lorna C Bautista, Gladi Lopez-Valentin, Adrienne Woods, Tabitha Jackson, Casey Chen, E Lee Poythress, Sharon Venus

**Affiliations:** Baylor College of Medicine, Houston, TX; Harris Health, Houston, Texas; Harris Health, Houston, Texas; Harris Health, Houston, Texas; Harris Health, Houston, Texas; Harris Health, Houston, Texas; Harris Health, Houston, Texas; Harris Health, Houston, Texas; Harris Health, Houston, Texas; Baylor College of Medicine, Houston, TX; Harris Health, Houston, Texas

## Abstract

**Background:**

Diabetic foot ulcers (DFU) are the leading cause of non-traumatic lower extremity amputations in the United States, despite being largely treatable with proper care. Patients at Ben Taub Osteomyelitis Wound Clinic (BTOWC) face obstacles in managing their DFUs due to limited health literacy, financial constraints, and language barriers. From January to July 2021, the average 90-day wound healing rate at BTOWC was 26.3%, significantly lower than the 40-50% rate typically reported in literature. This project was performed to improve 90-day wound healing rate for DFU patients at BTOWC from 26.3% to 40% over 3 years.

**Figure:**
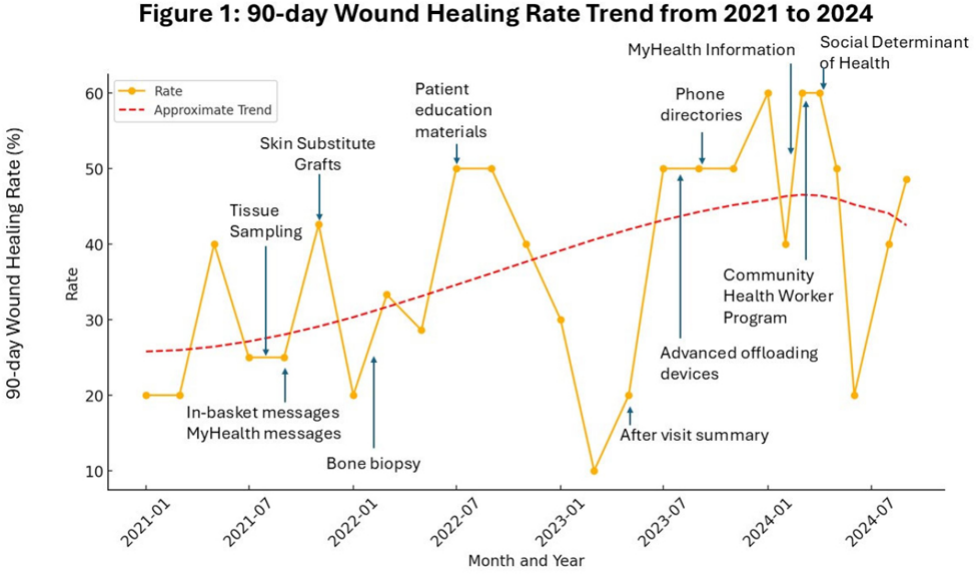

**Methods:**

The primary measure was 90-day wound healing rate for DFU patients at BTOWC. The team members included BTOWC physicians, therapists, technicians, front desk staff and case managers. BTOWC implemented several interventions: 1) Expanded in-clinic procedures, including tissue sampling for microbiology diagnosis, bone biopsies, and skin substitute grafts, 2) Provided paper-based information such as phone directories, patient education materials, and patient portal information, 3) Enhanced collaboration between providers and case management to address social determinants of health and to improve diabetes control through a community program, 4) Improved utilization of electronic medical records for better communication among providers and with patients such as in-basket messages and after visit summary, 5) Made advanced offloading devices available through a grant. Epic Slicer Dicer was used for data collection. One tailed t-test was used for statistical analysis.

**Results:**

This project involved 1107 DFU patients who had new appointments at BTOWC from January 2021 to October 2024. After excluding 220 patients, chart reviews were performed on 294 patients. Results showed a trend of improved wound healing rates over the three- year period (Figure 1), with the 90-day wound healing rate increasing from 26.3% to 41.8% (p-value .037).

**Conclusion:**

The multifaceted approach involving expanded in-clinic procedures, improved patient information, better case management collaboration, and enhanced use of electronic health records proved effective in improving wound healing rates for DFU patients facing socioeconomic challenges.

**Disclosures:**

Yuriko Fukuta, MD, PhD, CWSP, Elsevier: Honoraria

